# The heart's ‘little brain’ controlling cardiac function in the rabbit

**DOI:** 10.1113/expphysiol.2014.080168

**Published:** 2014-10-29

**Authors:** Kieran E Brack

**Affiliations:** 1 Department of Cardiovascular Sciences, Cardiology Group, Glenfield Hospital, University of LeicesterUK; 2 Leicester NIHR Biomedical Research Unit in Cardiovascular Disease, Glenfield HospitalLeicester, UK

## Abstract

New Findings

•What is the topic of this review?

The topic of the review is the intrinsic cardiac nervous system in the rabbit.

•What advances does it highlight?

The anatomy of rabbit intrinsic ganglia is similar to that of other species, including humans. Immunohistochemistry confirms the presence of cholinergic and adrenergic neurones, with a striking arrangement of neuronal nitric oxide synthase-positive cell bodies. Activation of atrial ganglia produces effects on local and remote regions.

Heart disease is a primary cause of mortality in the developed world, and it is well recognized that neural mechanisms play an important role in many cardiac pathologies. The role of extrinsic autonomic nerves has traditionally attracted the most attention. However, there is a rich intrinsic innervation of the heart, including numerous cardiac ganglia (ganglionic plexuses), that has the potential to affect cardiac function independently as well as to influence the actions of the extrinsic nerves. To investigate this, an isolated, perfused, innervated rabbit Langendorff heart preparation was considered the best option. Although ganglionic plexuses have been well described for several species, there was no full description of the anatomy and histochemistry of rabbit hearts. To this end, rabbit intrinsic ganglia were located using acetylcholinesterase histology (*n* = 33 hearts). This revealed six generalized ganglionic regions, defined as a left neuronal complex above the left pulmonary vein, a right neuronal complex around the base of right cranial vein, three scattered in the dorsal right atrium and a region containing numerous ventricular ganglia located on the conus arteriosus. Using immunohistochemistry, neurons were found to contain choline acetyltransferase or tyrosine hydroxylase and/or neuronal nitric oxide synthase in differing amounts (choline acetyltransferase > tyrosine hydroxylase > neuronal nitric oxide synthase). The function of rabbit intrinsic ganglia was investigated using a bolus application of nicotine or electrical stimulation at each of the above sites whilst measuring heart rate and atrioventricular conduction. Nicotine applied to different ganglionic plexuses caused a bradycardia, a tachycardia or a mixture of the two, with the right atrial plexus producing the largest chronotropic responses. Electrical stimulation at these sites induced only a bradycardia. Atrioventricular conduction was modestly changed by nicotine, the main response being a prolongation. Electrical stimulation produced significant prolongation of atrioventricular conduction, particularly when the right neuronal complex was stimulated. These studies show that the intrinsic plexuses of the heart are important and could be crucial for understanding impairments of cardiac function. Additionally, they provide a strong basis from which to progress using the isolated, innervated rabbit heart preparation.

## Introduction

Historically, interpretation of autonomic cardiotropic effects was confined to centrally derived extrinsic inputs from sympathetic excitatory and parasympathetic inhibitory nerves. However, it has become apparent that neurocardiac control is more complex owing to a network of intrinsic neurones constituting numerous plexuses and ganglia spread widely over the cardiac chambers, giving rise to the idea of a ‘little brain’ influencing cardiac function (Ardell, 2004).

## Anatomy of intrinsic cardiac innervation

Extrinsic autonomic nerves access the heart along arterial routes and extend from the atria directly onto the ventricular epicardium. Despite early observations of ganglia around the coronary sinus and nodal areas, it has only recently been fully realized that as well as supplying the sinoatrial and atrioventricular nodes, extrinsic nerves may also synapse with cell bodies of intrinsic cardiac nerves. The majority of intrinsic nerve cell bodies reside on supraventricular tissues, lying flat on the epicardial surface and also within fat pads, with axons forming connections with other nearby neurones, thus giving rise to networks that appear as ‘ganglionic plexuses’ (GPs). The widespread distribution of these ganglia and the extensive networks of the plexuses suggest that intrinsic nerves play an important role in cardiac function.

To study the physiology of the GPs and their interaction with extrinsic nerves, I have used the isolated, innervated Langendorff rabbit heart preparation (Ng *et al*. 2001; Brack, 2003), because the extrinsic autonomic nerves are intact but isolated from the central nervous system, and blood-circulating agents can be controlled precisely. However, while the location of ganglia has been described in many experimental animals as well as in humans (Yuan *et al*. 1994; Horackova *et al*. 1999; Leger *et al*. 1999; Pauza *et al*. 2000; Richardson *et al*. 2003; Batulevicius *et al*. [Bibr b5],[Bibr b6]; Sabukina, 2010; Rysevaite *et al*. 2011), there was no description in the rabbit. Therefore, using pressure-distended whole hearts and whole-mount preparations from 33 rabbits, the topography of the GPs on the heart surface and interatrial septum was demonstrated using the pan-neuronal technique of acetycholinesterase histology (Sabukina *et al*. 2014).

Intrinsic nerves are usually grouped into defined subplexus routes projecting to different effector sites, with between five and seven subplexuses that are species dependent. In the rabbit, nerves approaching the heart extended epicardially and innervated the atria, interatrial septum and ventricles by five nerve subplexuses, i.e. left and middle dorsal, dorsal right atrial, ventral right and left atrial subplexuses. Numerous extrinsic nerves accessed the arterial part of the heart hilum between the aorta and pulmonary trunk and innervated the ventricles by the left and right coronary subplexuses. Somata of intrinsic neurons, typically 15–30 and 20–45 μm in size on the short and long axis (Leger *et al*. 1999; Rysevaite *et al*. 2011), respectively, occur as individual entities or gathered into ganglia or grouped into ‘clusters’. In the rabbit, over 2200 such intrinsic neurons were found in six regions, generalized into left and right neuronal complexes (LNC and RNC, respectively). The LNC contained the largest number of intrinsic neurons (∼1200) in the following three localities: (i) at the root of the left pulmonary vein; (ii) between the left and middle pulmonary vein; and (iii) between the middle pulmonary vein and caval vein. The RNC contained ∼800 neurons, also in three locations, as follows: (i) the dorsocranial groove above the interatrial septum; (ii) on the ventral right atrial region around the base of right cranial vein, extending to and around the right pulmonary vein; with (iii) some RNC ganglia scattered on the dorsal right atrium, extending onto the tail of the sinoatrial node. Finally, cell bodies from ∼45 intrinsic nerves were found in ventricular ganglia that were located on the conus arteriosus. Thin commissural nerves were identified to connect the left and right neuronal clusters in the heart hilum and also between the left and right coronary subplexuses in the ventricle (Saburkina *et al*. 2014), suggesting that some of these nerves might represent local circuit neurons. Despite anatomical differences in the distribution of intrinsic cardiac neurons and the presence of a well-developed nerve plexus within the heart hilum, the topography of all seven subplexuses of the intrinsic nerve plexus in the rabbit heart corresponds well with that of other mammalian species, including humans. However, the number of cardiac ganglia is highly species dependent, ranging from 19 in the mouse (Rysevaite *et al*. 2011) to 700 in humans, where they are more scattered (Pauza *et al*. 2000). Ganglia are heterogeneously innervated by bilateral autonomic inputs, but I am in the process of confirming this in the rabbit.

## Neurochemical phenotype

Historically, it was thought that intrinsic ganglia were simple relay stations for parasympathetic inputs and, by assumption, would contain only cholinergic markers, e.g. choline acetyltransferase (ChAT), the enzyme responsible for the synthesis of acetylcholine, thereby suggesting that all ganglionic somata would be parasympathetic postganglionic efferents. We recently tested this theory in rabbit ganglia by performing immunohistochemistry on microdissected rabbit whole-atrial preparations. Primary antibodies were raised against the neurotransmitters ChAT, tyrosine hydroxylase (TH) and neuronal nitric oxide synthase (nNOS) from seven hearts. Choline acetyltransferase-positive neurons were found in the largest quantity, with 2187 ± 396 somata (range 1733–2976) identified in 93 ± 4 ganglia (range 86–99). Tyrosine hydroxylase-positive cell bodies were the second most abundant, with 1157 ± 546 neurons (range 611–1702) found in 38 ± 0 ganglia (range 38–38). Neuronal nitric oxide synthase-containing cell bodies were the least populous, with 205 ± 111 neurons (range 95–315) discovered in 31 ± 21 ganglia (range 10–51). As expected, ChAT-positive cell bodies were diffusely distributed across the whole heart within every ganglionic location, which is in accord with data from my acetylcholinesterase study (Saburkina *et al*. 2014).

The largest groups of TH-containing neurons were found within the heart hilum in ganglia containing between 20 and 65 TH-immunoreactive cell bodies that were primarily in the right neuronal cluster. Smaller groups of TH-positive neurons (6–10 cells) were diffusely located in the remaining cardiac ganglia, particularly in the right atrial ganglionic plexus, which innervates the sinoatrial node influentially. Singular TH-containing neurons and ganglia of one to five cells were located around the bases of the pulmonary veins/caval vein. Tyrosine hydroxylase-positive neurones were categorized into the following two populations: (i) TH-immunoreactive cell bodies only immunoreactive for TH, suggesting that they were pure, presumably postganglionic, ‘sympathetic’ nerves; and (ii) TH co-localized within neurons that are also immunoreactive for ChAT, i.e. biphenotypic. In ganglia with both neurotransmitters present, the degree of co-localization of ChAT and TH in ganglia containing between five and 300 neurones ranged from 33 to 100% for larger and smaller ganglia, respectively.

The most striking result of this study was the topographical arrangement of nNOS neurons. Neuronal nitric oxide synthase immunoreactivity was found in ganglia that were usually small in size, containing up to 20 neurons, concentrated around the bases of the left, middle and right pulmonary vein, and a small number of ganglia spreading towards the dorsal right atrium, overlapping the tail of the sinoatrial node. Neuronal nitric oxide synthase-positive neurones were typically associated with cell bodies also immunoreative for ChAT. There was a wide variation of co-localization of ChAT and nNOS within cardiac ganglia. Most ChAT-immunoreactive ganglia, typically containing <12 cell bodies, were usually devoid of nNOS. In ganglia containing more than an average of 12 cell bodies, the numbers of ChAT-positive cell bodies co-labelled with nNOS ranged from 16 to 75%.

These data are in accord with a growing body of evidence that continues to redefine and expand the range of neurochemicals present in the intracardiac ganglia of other species; these include vasoactive intestinal peptide (known to be co-released alongside Ach; Kuncova *et al*. 2003), neuropeptide Y (known to be co-released alongside noradrenaline; Richardson *et al*. 2003), synaptophysin (a marker of presynaptic fibres; Richardson *et al*. 2003), substance P (Rysevaite *et al*. 2011) and calcitonin gene-related peptide (Rysevaite *et al*. 2011). Both substance P and calcitonin gene-related peptide are considered as markers of afferent terminals. Cardiac ganglia are therefore complex structures containing efferent pre- and postganglionic parasympathetic nerves, presumed postganglionic sympathetic nerves and a physiologically undefined bi-phenotypic subpopulation that co-localize with cholinergic markers, all of which are innervated by sensory nerves.

## Physiology of the heart's ‘little brain’

The foregoing description implies that the physiological actions of intrinsic ganglia could have a considerable effect on cardiac function. I have tested this using the non-innervated, Langendorff-perfused rabbit heart (Patel *et al*. 2008), using a small bolus of nicotine or electrical stimulation applied to the following four regions containing ganglia: (i) at the root of the left pulmonary vein (left neuronal complex; LNC); (ii) on the medial aspect of the right cranial vein (right neuronal cluster, RNC); (iii) at the junction between the middle pulmonary vein and caudal vein region; and (iv) at the dorsal right atrial region (RAGP). Heart rate (HR) and atrioventricular (AV) conduction (AVC) were measured. Chronotopic effects from nicotine applied to each region were classified as a pure bradycardia, a pure tachycardia or a mixture of both responses. Heart rate responses of each category were seen at each region where nicotine was applied, including that of the left pulmonary vein. The bradycardic and tachycardic response was largest when nicotine was applied to the RAGP region, suggesting that this region strongly innervates the sinoatrial node. In contrast, electrical stimulation of LNC or RNC only decreased HR, with much larger responses from the RNC. All bradycardias were blocked by atropine whilst all tachycardias were blocked by metoprolol, indicating that cholinergic and adrenergic mechanisms, respectively, were most probably responsible for the changes in HR. Furthermore, all responses to electrical stimulation were prevented using hexamethonium, a nicotinic acetylcholine receptor blocker, indicating that these changes were mediated through ganglionic mechanisms.

Atrioventricular conduction was measured in sinus rhythm and during constant atrial pacing by recording the interval from the atrial electrogram to a ventricular monophasic action potential recorded from the left ventricle. During sinus rhythm, changes in AVC were difficult to interpret because of the intrinsic rate-dependence property of the AV node, e.g. AVC decreases (seen as a prolongation in AV interval) with increases in HR. Despite this, it was clear that only rate-dependent changes in AVC were seen when nicotine was applied to the RAGP. During constant atrial pacing, RAGP stimulation did not elicit any changes in AVC, confirming little or no innervation of the AV node from these ganglia in the rabbit. From the three remaining stimulation sites, nicotine applied to the junction between the middle pulmonary vein and caudal vein region produced only an increase in AV interval (suggesting AV delay), whilst both increases and decreases in AV interval (AV delay and AVC shortening, respectively) were seen following LNC and RNC stimulation. The prolongation in AV interval was largest from the RNC, whilst AV interval shortening was equipotent at LNC and RNC stimulation sites. Atropine and metoprolol abolished all increases and decreases in AV interval, suggesting that cholinergic and adrenergic signalling pathways mediated these responses. Hexamethonium abolished all responses, showing that they were dependent on ganglionic presynaptic activation. These data are in accord with studies using nicotine (Cardinal *et al*. 2009) or direct electrical stimulation (Butler *et al*. 1990) of hearts *in situ* in the dog.

## Interaction with extrinsic nerves

An important feature of GP physiology is their role during and interaction with peripheral autonomic nerve activity. Recently, intracellular recordings made from postganglionic vagal neurons in the right atrium (McAllen *et al*. 2011) have shown that GPs can determine the level of postganglionic output. However, questions remain regarding the influences of other GPs on extrinsic parasympathetic or sympathetic nerves. Our studies in the *in vitro* innervated rabbit heart consolidate knowledge that there is a high degree of lateralization in the effects of sympathetic (Winter *et al*. 2012) and vagus nerve stimulation (Ng *et al*. 2001) on cardiodynamics, suggesting that these heterogeneous effects could also reflect selective GP involvement, and there are some data to support this.

We have shown that electrical stimulation of sympathetic inputs increases whilst electrical stimulation of the cervical vagus nerve decreases the susceptibility of the heart to ventricular fibrillation (Ng *et al*. 2007). Studies in the *in vitro* innervated heart suggest that the following two separate electrophysiological cardiac mechanisms mediate this protection: (i) a muscarinic effect on heart rate and ventricular effective refractory period (Brack *et al*. 2011); and (ii) a reduction in electrical restitution slope that is independent from the actions of acetylcholine and is mediated by NO (Brack *et al*. 2007). Vagal protection was abolished in the presence of hexamethonium, confirming that intrinsic ganglia are involved in the effects of cervical vagus nerve stimulation and suggesting that they involve a select population of postganglionic nitrergic nerves. This possibility was strengthened by our identification of nNOS-positive neurones in the immunohistochemical study mentioned above.

In the dog, recent work suggests that protection from ventricular arrhythmia may involve the left superior and anterior right atrial GPs. Individual or simultaneous stimulation of these ganglia increases the ventricular action potential whilst reducing the electrical restitution slope (He *et al*. 2013), similar to our studies in rabbits (Ng *et al*. 2007; Brack *et al*. [Bibr b12],[Bibr b9]), which was associated with a lower incidence of electrical alternans, suggesting a reduced propensity for normal hearts to ventricular arrhythmia. Interestingly, chemical stimulation of the right atrial GP neurons in the dog also induces ventricular arrhythmia (Huang *et al*. 1994). Clearly, the role of the intrinsic cardiac nervous system in ventricular arrhythmia is not understood, and further research is needed.

Previous studies on anaesthetized dogs using hexamethonium applied to specific GPs *in situ* suggest that they can have significant effects on vagal control (Gatti *et al*. 1995; Dickerson *et al*. 1998; Gray *et al*. 2004). This is in accord with my studies in the rabbit, which have shown that the effect of vagal stimulation is enhanced following nicotinic enhancement of atrial GPs (Brack *et al*. 2012). The studies on the rabbit described above extended to the innervated, isolated rabbit heart preparation will enable us to test more quantitatively the interaction of the GPs at particular sites in the heart with parasympathetic control of a range of cardiac functions.

Concerning adrenergic control, the often-overlooked discovery of TH-containing somata in most intrinsic ganglia, including the data I have generated in the rabbit, could redefine the nature of cardiac sympathetic innervation. We previously showed that left- and right-sided sympathetic inputs differentially affect heart rate, atrioventricular condition, left ventricular force and left ventricular electrophysiology (Winter *et al*. 2012). Further work activating GPs is warranted to clarify whether any of the previously found TH-immunoreactive intrinsic neurones are involved in these sympathetic nerve effects.

## Sympathovagal interaction

The most recognized form of interaction between the cardiac effects of sympathetic and parasympathetic nerves is ‘accentuated antagonism’, whereby the inhibitory effects of the vagus nerve are augmented in the presence of sympathoexcitation. Other interactions are known to occur, such as the inhibition of vagal effects by the sympathetic nerves. While this is the case, sympathovagal interaction in the heart has historically concentrated on the reciprocal modulation of acetylcholine and noradrenaline release from postsynaptic nerve terminals at the neuroeffector junction and/or interaction of postreceptor signalling pathways involving cAMP and cGMP systems. This presence of TH-containing cell bodies within a variety of intrinsic ganglia, which are thought to have regional cardiac effects, raises the possibility that there is an additional, previously unknown level of interaction between the cholinergic and adrenergic systems, i.e. at the level of the cardiac ganglia.

## Concluding remarks

Owing to the pioneering work by Armour and colleagues, there is growing recognition of the functional capabilities of GPs. However, the full extent to which GPs modify cardiac performance and pathology remains unclear, and information pertaining to functional connectivity between plexuses or the extent to which the intrinsic cardiac nervous system can affect regional actions of extrinsic autonomic nerves is needed. More importantly, there are several features of cardiac disease that involve altered autonomic control, including atrial and ventricular arrhythmia (Brack & Ng, 2014), myocardial ischaemia and heart failure (Brack *et al*. 2012). Recently, it has been shown that atrial GP neurons are hypertrophied following heart failure (Singh *et al*. 2013), providing evidence of anatomical changes following cardiac disease. These changes would be expected to affect neuronal cell communication, detrimentally affecting ganglionic physiology, and could, in part, explain the attenuation in GP neurotransmission following heart failure (Bibevski & Dunlap, 1999). Understanding how GPs are involved in other disease situations could be crucial to reverse pathological autonomic imbalance. As stated by Jeff Ardell (2004), ‘That the cardiac milieu is transduced at the level of the heart independent to central control requires a thorough reassessment … taking into account of how the cardiac neuronal hierarchy responds to altered cardiovascular status, one may be able to ascertain how neuronal substations adapt in pathology’.

## Call for comments

Readers are invited to give their opinion on this article. To submit a comment, go to: http://ep.physoc.org/letters/submit/expphysiol;100/4/348

## References

[b1] Ardell JL, Ardell JL, Armour JA (2004). Intrathoracic neuronal regulation of cardiac function. Basic and Clinical Neurocardiology.

[b2] Armour JA (1996). Histamine-sensitive intrinsic cardiac and intrathoracic extracardiac neurons influence cardiodynamics. Am J Physiol Regul Integr Comp Physiol.

[b3] Armour JA (1997). Intrinsic cardiac neurons involved in cardiac regulation possess alpha 1-, alpha 2-, beta 1- and beta 2-adrenoceptors. Can J Cardiol.

[b4] Armour JA, Huang MH, Smith FM (1993). Peptidergic modulation of in situ canine intrinsic cardiac neurons. Peptides.

[b5] Batulevicius D, Pauziene N, Pauza DH (2005). Architecture and age-related analysis of the neuronal number of the guinea pig intrinsic cardiac nerve plexus. Anat Rec.

[b6] Batulevicius D, Skripka V, Pauziene N, Pauza DH (2008). Topography of the porcine epicardiac nerve plexus as revealed by histochemistry for acetylcholinesterase. Auton Neurosci.

[b7] Bibevski S, Dunlap ME (1999). Ganglionic mechanisms contribute to diminished vagal control in heart failure. Circulation.

[b8] Brack KE (2003).

[b9] Brack KE, Coote JH, Ng GA (2011). Vagus nerve stimulation protects against ventricular fibrillation independent of muscarinic receptor activation. Cardiovasc Res.

[b10] Brack KE, Coote JH, Ng GA (2012). Modulation if vagus nerve stimulation via ganglionic plexus activation and ventricular ischemia. FASEB J.

[b11] Brack KE, Tintoiu IC, Ng GA, Kibod AS, Knight BP, Essebag V, Fishberger SB, Slevin M (2014). Autonominc control of cardiac arrhythmia. Cardiac Arrhythmias: from Basic Mechanisms to State-of-the-Art Management.

[b12] Brack KE, Patel VH, Coote JH, Ng GA (2007). Nitric oxide mediates the vagal protective effect on ventricular fibrillation via effects on action potential duration restitution in the rabbit heart. J Physiol.

[b13] Butler CK, Smith FM, Cardinal R, Murphy DA, Hopkins DA, Armour JA (1990). Cardiac responses to electrical stimulation of discrete loci in canine atrial and ventricular ganglionated plexi. Am J Physiol Heart Circ Physiol.

[b14] Cardinal R, Page P, Vermeulen M, Ardell JL, Armour JA (2009). Spatially divergent cardiac responses to nicotinic stimulation of ganglionated plexus neurones in the canine heart. Auton Neurosci.

[b15] Chang HY, Lo LW, Lin YJ, Lee SH, Chiou CW, Chen SA (2014). Relationship between intrinsic cardiac autonomic ganglionated plexi and the atrial fibrillation nest. Circ J.

[b16] Dickerson LW, Rodak DJ, Fleming TJ, Gatti PJ, Massari VJ, McKenzie JC, Gillis RA (1998). Parasympathetic neurons in the cranial medial ventricular fat pad on the dog heart selectively decrease ventricular contractility. J Auton Nerv Syst.

[b17] Gatti PJ, Johnson TA, Phan P, Jordan IK, Coleman W, Massari VJ (1995). The physiological and anatomical demonstration of functionally selective parasympathetic ganglia located in discrete fat pads on the feline myocardium. J Auton Nerv Syst.

[b18] Gray AL, Johnson TA, Ardell JL, Massari VJ (2004). Parasympathetic control of the heart. II. A novel interganglionic intrinsic cardiac circuit mediates neural control of heart rate. J Appl Physiol.

[b19] He B, Lu Z, He W, Huang B, Yu L, Wu L, Cui B, Hu X, Jiang H (2013). The effects of atrial ganglionated plexi stimulation on ventricular electrophysiology in a normal canine heart. J Interv Card Electrophysiol.

[b20] Horackova M, Armour JA, Byczko Z (1999). Distribution of intrinsic cardiac neurons in whole-mount guinea pig atria identified by multiple neurochemical coding. A confocal microscope study. Cell Tissue Res.

[b21] Huang MH, Wolf SG, Armour JA (1994). Ventricular arrhythmias induced by chemically modified intrinsic cardiac neurones. Cardiovasc Res.

[b22] Kuncová J, Slavíková J, Reischig J (2003). Distribution of vasoactive intestinal polypeptide in the rat heart: effect of guanethidine and capsaicin. Ann Anat.

[b23] Leger J, Croll RP, Smith FM (1999). Regional distribution and extrinsic innervation of intrinsic cardiac neurons in the guinea pig. J Comp Neurol.

[b24] McAllen RM, Salo LM, Paton JFR, Pickering AE (2011). Processing of central and reflex vagal drives by rat cardiac ganglion neurones: an intracellular analysis. J Physiol.

[b25] Momose M, Tyndale-Hines L, Bengel FM, Schwaiger M (2001). How heterogeneous is the cardiac autonomic innervation. Basic Res Cardiol.

[b26] Ng GA, Brack KE, Coote JH (2001). Effects of direct sympathetic and vagus nerve stimulation on the physiology of the whole heart – a novel model of isolated Langendorff perfused rabbit heart with intact dual autonomic innervation. Exp Physiol.

[b27] Ng GA, Brack KE, Patel VH, Coote JH (2007). Autonomic modulation of electrical restitution, alternans and ventricular fibrillation initiation in the isolated heart. Cardiovasc Res.

[b28] Patel VH, Brack KE, Coote JH, Ng GA (2008). A novel method of measuring nitric-oxide-dependent fluorescence using 4,5-diaminofluorescein (DAF-2) in the isolated Langendorff-perfused rabbit heart. Pflugers Arch.

[b29] Pauza DH, Skripka V, Pauziene N, Stropus R (2000). Morphology, distribution, and variability of the epicardiac neural ganglionated subplexuses in the human heart. Anat Rec.

[b30] Richardson RJ, Grkovic I, Anderson CR (2003). Immunohistochemical analysis of intracardiac ganglia of the rat heart. Cell Tissue Res.

[b31] Rysevaite K, Saburkina I, Pauziene N, Vaitkevicius R, Noujaim SF, Jalife J, Pauza DH (2011). Immunohistochemical characterization of the intrinsic cardiac neural plexus in whole-mount mouse heart preparations. Heart Rhythm.

[b32] Saburkina I, Gukauskiene L, Rysevaite K, Brack KE, Pauza AG, Pauziene N, Pauza DH (2014). Morphological pattern of intrinsic nerve plexus distributed on the rabbit heart and interatrial septum. J Anat.

[b33] Saburkina I, Rysevaite K, Pauziene N, Mischke K, Schauerte P, Jalife J, Pauza DH (2010). Epicardial neural ganglionated plexus of ovine heart: anatomic basis for experimental cardiac electrophysiology and nerve protective cardiac surgery. Heart Rhythm.

[b34] Singh S, Sayers S, Walter JS, Thomas D, Dieter RS, Nee LM, Wurster RD (2013). Hypertrophy of neurons within cardiac ganglia in human, canine, and rat heart failure: the potential role of nerve growth factor. J Am Heart Assoc.

[b35] Winter J, Tanko AS, Brack KE, Coote JH, Ng GA (2012). Differential cardiac responses to unilateral sympathetic nerve stimulation in the isolated innervated rabbit heart. Auton Neurosci.

[b36] Yuan BX, Ardell JL, Hopkins DA, Losier AM, Armour JA (1994). Gross and microscopic anatomy of the canine intrinsic cardiac nervous system. Anat Rec.

